# Caesarean sections, indications and outcomes: a cross-sectional study using the Robson classification in a tertiary hospital in Sierra Leone

**DOI:** 10.1136/bmjopen-2023-081143

**Published:** 2024-09-03

**Authors:** Matteo Arata, Sonia Boyle, Beatrice Sgorbissa, Francesca Tognon, Valerie John-Cole, Michele Orsi, Claudia Caracciolo, Carlo Saccardi, Fabio Manenti, Giovanni Putoto, Abibatu K Kamara, Ana Pilar Betran

**Affiliations:** 1 Department of Women and Children's Health, University of Padua, Padova, Italy; 2 Department of Obstetrics and Gynecology Princess Christian Maternity Hospital (PCMH), University of Sierra Leone, Freetown, Sierra Leone; 3 Department of Cardiac Thoracic and Vascular Sciences and Public Health, University of Padua, Padua, Italy; 4 Doctors with Africa CUAMM, Padua, Italy; 5 College of Medicine and Allied Health Sciences, University of Sierra Leone, Freetown, Sierra Leone; 6 Mangiagalli Center, Fondazione IRCCS Ca' Granda Ospedale Maggiore Policlinico, Milan, Italy; 7 Government of Sierra Leone Ministry of Health and Sanitation, Freetown, Western Area, Sierra Leone; 8 Department of Sexual and Reproductive Health and Research, World Health Organization, Geneva, Switzerland

**Keywords:** OBSTETRICS, Maternal medicine, Public Hospitals, Cesarean Section

## Abstract

**Abstract:**

**Objective:**

WHO recommends the use of the Robson’s ‘Ten Groups Classification’ for monitoring and assessing caesarean section (CS) rates. The aim of this study was to investigate the rates, indications and outcomes of CS using Robson classification in a tertiary hospital in Sierra Leone.

**Design:**

Cross-sectional study.

**Setting:**

Princess Christian Maternity Hospital (PCMH), Freetown, Sierra Leone.

**Participants:**

All women who gave birth in PCMH from 1 October 2020 to 31 January 2021.

**Primary and secondary outcome measures:**

Primary outcome: CS rate by Robson group. Secondary outcomes: indications for CS and the newborn outcomes for each Robson group.

**Results:**

1998 women gave birth during the study period and 992 CS were performed, with a CS rate of 49.6%. Perinatal mortality was 7.8% and maternal mortality accounted for 0.5%. Two-thirds of the women entered labour spontaneously and were considered at low risk (groups 1 and 3). CS rates in these groups were very high (43% group 1 and 33% group 3) with adverse outcomes (perinatal mortality, respectively, 4.1% and 6%). Dystocia was the leading indication for CS accounting for about two-thirds of the CS in groups 1 and 3. Almost all women with a previous CS underwent CS again (95%). The group of women who give birth before term (group 10) represents 5% of the population with high CS rate (50%) mainly because of emergency conditions.

**Conclusion:**

Our data reveals a notably high CS rate, particularly among low-risk groups according to the Robson classification. Interpretation must consider PCMH as a referral hospital within an extremely low-resourced healthcare system, centralising all the complicated deliveries from a vast catchment area. Further research is required to assess the impact of referred obstetrical complications on the CS rate and the feasibility of implementing measures to improve the management of women with dystocia and previous CS.

**Strengths and limitations of this study**

- Indications for caesarean section (CS), outcomes of newborns and women were investigated within each Robson group for a better understanding of practices and possible needs.
- Birth registries did not report referral status, preventing in-depth analysis of the contribution of referral cases, accounting for almost one-third of admissions, to the CS rate.
- Only the type of health professional who conducted the CS was recorded. Information about the staff responsible for the decision for CSs was not reported, limiting further analysis and understanding of decision-making processes.
- Maternal and neonatal deaths occurring after discharge were not recorded.

## Introduction

 Caesarean section (CS) is a key surgical intervention to improve the outcome for the mother and fetus. The appropriate use of CS during childbirth is crucial: high rates may indicate unnecessary use of the intervention, while low rates may indicate unmet obstetrical needs and inadequate access to care.^1^,^3^

CS is not without risks so when a CS is performed without medical indication, it exposes women to unnecessarily increased risk of complications in the short-term (eg, blood loss, infections, visceral injury, thromboembolism, anaesthesia-related complications), in the long-term (pelvic adhesion, chronic pain, sexual dysfunction, subfertility) and for future pregnancies (eg, placental problems, uterine rupture, stillbirth and preterm birth).^4^,^8^ These risks are exacerbated in women in low-resource settings where lack of medical equipment, inadequately trained personnel or limited access to health facilities can lead to suboptimal management of complications.^1 4^

In 1985, the WHO suggested the appropriate CS rate to be 10–15% at the population level.^9^ Since then, CS rates have increased globally to unprecedented levels^3 10^ raising concern about the consequences of this increase. Since 2015, the WHO has not endorsed an ‘ideal’ CS rate but rather emphasises the need to monitor CS rates in a meaningful, reliable and action-oriented manner at the healthcare facility level.^11^ For this purpose, WHO recommends the Ten Group Classification, also known as Robson classification^12^ as a global standard for assessing, monitoring and comparing CS rates.^11^

The Robson classification can be used to study and assess CS rates in more uniform groups of women and in relation to other perinatal outcomes and processes. More targeted interventions can be designed and implemented in each group independently and subsequently evaluated. The classification has been successfully used to identify both overuse and underuse in high-, low- and middle-income countries, thus showing value across a variety of resource levels.^13^

Sierra Leone has one of the highest maternal mortality ratio, stillbirth and neonatal death rate worldwide. Strategies implemented by the governments and partners in the country to reduce mortality have aimed to increase access to skilled attendants at birth and emergency obstetrical care, including CS.^14^ In Sierra Leone, the CS rate at the national level remains low, although the country has witnessed a rapid increase in the last two decades, from 1.5% (2003–2008) to 3% (2012–2017).^3^ The quality of care for surgery remains suboptimal, with a high perioperative mortality rate of 1.5% and haemorrhage, hypertensive disorders and sepsis being the most important causes of death in women undergoing a CS.^15 16^ Nevertheless, the use of CS varies widely between districts^15^ showing that disparities in the healthcare assistance coexist.

The aim of this study was to investigate the rate of CS, the indications, as well as maternal and newborns’ outcomes at Princess Christian Maternal Hospital in Freetown, Sierra Leone, using the Robson classification. An additional objective was to identify substandard practices and recommend strategies aimed at improving maternity care.

## Methods

### Study design

We conducted a cross-sectional study at the Princess Christian Maternity Hospital, in Freetown (Western Area Urban District), Sierra Leone. Our study population consisted of all women who gave birth in this hospital during a 4-month period between 1 October 2020 and 31 January 2021.

We used the Robson classification system to study women. The Robson system classifies all women admitted for birth in the facility on the basis of essential obstetrical characteristics which are routinely collected in all maternities for the clinical care of the women (parity, gestational age, number of fetuses, previous CS, on-set of labour and fetal presentation and lie). We used the WHO Implementation Manual on the Robson classification system as the main guide for the analysis and interpretation.^17^

### Context

The Princess Christian Maternity Hospital (PCMH) is a tertiary-level government hospital located in Sierra Leone’s capital city, Freetown. It is the main referral hospital for maternal care in the capital’s Western Area district, with a catchment area of about 1 million inhabitants.

Healthcare for mothers and newborns in Sierra Leone is provided free of charge according to the Ministry of Health and Sanitation (MoHS) national ‘Free Health Care Initiative’.^18 19^ However, the availability of drugs and consumables is irregular and additional financial support such as donations, is essential. Doctors with Africa CUAMM is a non-governmental organisation collaborating with the MoHS and supporting PCMH since 2016 as the main technical partner.

Every year, the hospital admits about 9000 patients to the maternity ward, attends more than 6000 births, provides more than 20 000 antenatal visits and 18 000 outpatient visits for women and children, as well as comprehensive care services for obstetrical emergencies.^20^ The hospital has 119 beds and includes various wards and units: general maternity, eclamptic, puerperium, postoperative, antenatal and gynaecology. The hospital employs nurses, midwives and four teams each consisting of one obstetrician/gynaecologist, one medical officer (licensed non-specialist physician), some junior doctors in quarterly rotation and one surgical community health officer, who is a staff trained to perform surgical practices such as CS, under MoHS regulation.

### Variables, data collection and analysis

PCMH does not have a digitalised system for medical records. The presence of two dedicated volunteer doctors facilitated data collection during the study period. Data were obtained from patient’s paper charts, labour ward and operating theatre registers, and were entered by members of the research team in a Microsoft Excel database specifically designed for this study.

Variables collected included maternal age, obstetrical history (parity, previous CS, fetal presentation), mode of birth (ie, spontaneous vaginal birth, operative vaginal birth or CS), the onset of labour (spontaneous, induced, pre-labour CS), staff who performed the CS (ie, obstetrician or surgical technicians) and the indication for CS. Moreover, we collected maternal and neonatal outcomes: maternal death, live birth, fresh stillbirth, macerated stillbirth, early neonatal death, Apgar score at 5 min.

For each woman who underwent a CS, a single indication was reported from the hospital registry. Diagnosis and definition of all pathological conditions were derived from the National Protocol and Guidelines for Emergency and Newborn Care.^21^ The accuracy of the indications assigned in relation to the definitions was not verified by the data collectors. When more than one indication was recorded, we selected only one for the analysis, according to a predefined hierarchy devised for this study based on earlier proposals in the literature:^22^,^25^ (1) Urgent or emergency CS (severe hypertensive disorder including severe pre-eclampsia, eclampsia, antepartum haemorrhage due to abruptio placentae or placenta previa, laparotomy for uterine rupture), (2) previous CS, (3) mechanical or dynamic dystocia (obstructed and prolonged labour, cephalopelvic disproportion, transverse lie, failed induction), (4) intrapartum acute fetal distress (including cord prolapse), (5) breech presentation, (6) maternal medical causes (severe anaemia, sickle cell disease, severe malaria) (7) fetal causes other than fetal distress (macrosomia, idrocefalo, twins, intrauterine fetal death) and (8) others (elective CS, unknown, post-date, prolonged premature rupture of membranes).

For multiple pregnancies, data were registered only for the twin born first. We used birth weight >2500 g as a proxy for gestational age >37 weeks in the Robson classification. This adaptation has been suggested and previously used for the Robson classification in settings where the accurate assessment of gestational age is a challenge.^23 26^

The maternal mortality rate was defined as the number of maternal deaths over the total number of live births. Stillbirth was defined as a baby born with no signs of life after 28 weeks of gestation or weighing more than 1000 g. We defined early neonatal death as the death of a live-born neonate, by discharge or day 7 of life whichever occurs first. Perinatal mortality was defined as the sum of stillbirths and early neonatal deaths among all deliveries.^27^ The CS rate was defined as the total number of caesarean deliveries among women divided by the total number of deliveries.^3^ The onset of labour was defined as regular contractions of at least three every 10 min, 100% effaced cervix and at least 4 cm dilatation.^28^

For the Robson classification, we calculated the total number and rate of CS in each of the 10 groups and both the absolute and relative contribution of each group to the total CS rate, maternal death and newborn outcomes (ie, live births, stillbirths, early neonatal deaths and newborns with an Apgar score <7 at 5 min discharged alive). We reported the indications for CS in each Robson group as the percentage of the total CS conducted in each group.^17^ We also report women with incomplete information on any obstetrical variables that prevented classification within one of the Robson groups. We used the threshold for fetal viability at a birth weight <1000 g and <28 weeks’ gestational age. Below this threshold, women were not included.

### Patient and public involvement

No patients/members of the public were involved in the definition of the research question or outcome measures, nor in the design and implementation of the study. We have no plans to involve patients/members of the public in the dissemination of the study’s results.

## Results

From 1 October 2020 to 31 January 2021, 1998 women delivered at the PCMH and 23 were unclassifiable in the Robson groups due to missing data. The characteristics of women and newborns are summarised in table 1.

**Table 1:** Characteristics of mothers and newborns, 1 October 2020 to 31 January 2021, Princess Christian Maternity Hospital, Sierra Leone (n=1998)

Maternal characteristics (n=1998)		
Age	Range	12–45
	Media	26
Parity	Parity=0	746 (37.4%)
	Parity=1	630 (31.5%)
	Parity ≥2	618 (30.9%)
	Missing	4 (0.2%)
Onset of labour	Spontaneous	1680 (84.1%)
	Induced	62 (3.1%)
	Pre-labour CS	242 (12.1%)
	Missing	14 (0.7%)
Previous CS	Yes	198 (10%)
	No	1792 (89.7%)
	Missing	8 (0.4%)
Number of fetuses	Single	1894 (94.8%)
	Multiple	101 (5%)
	Missing	3 (0.2%)
Presentation/lie	Cephalic	1849 (92.5%)
	Breech	124 (6.2%)
	Oblique/transverse	22 (1.1%)
	Missing	3 (0.2%)
Final mode of birth	Vaginal	990 (49.6%)
	Assisted vaginal*	12 (0.6%)
	Caesarean section	992 (49.6%)
	Missing	4 (0.2%)
CS operator (n=992)	Obstetrician/gynaecologist	287 (28.9%)
	Surgical technicians	705 (71.1%)
Maternal death		9 (0.5%)
Newborn characteristics		
Birth weight	<2.5 kg	155 (7.8%)
	≥2.5 kg	1837 (91.9%)
	Missing	6 (0.3%)
Newborns outcome	Live births	1838 (92.0%)
	Stillbirths	148 (7.4%)
	Early neonatal death	8 (0.4%)
	Missing	4 (0.2%)
Apgar score at 5 min among live births	<7	294 (16.0%)
	≥7	1544 (83.8%)
	Missing	4 (0.2%)

* Forceps or vacuum.

CScaesarean section

The average age of the study population was 26 years and about half of women (n. 992) gave birth by CS during the study period. More than 60% of women had one or more births previously, only 10% had a previous CS. The majority of the births (1849; 92.5%) were cephalic presentations. Onset of labour was spontaneous in 84.1% (1680) of women while only 3.1% (62) were induced. Surgical technicians performed 71.1% (705) of all CS. Regarding neonatal outcomes, 148 (7.4%) stillbirths and 8 (0.4%) early neonatal deaths have been reported with a perinatal mortality rate of 7.8%. Forceps or vacuum was used to assist birth in 12 (0.6%) women.

Table 2 shows the Robson classification including outcomes by group. Most women were in group 3, comprising multiparous women with a single-term pregnancy in spontaneous labour (38.6% of the study population) and group 1, consisting of nulliparous women with a single-term pregnancy in spontaneous labour (28.3%). Group 5 included 8.6% of the population. Groups 1 and 3, which are normally considered to be at low risk of CS, presented a CS rates of 42.6% and 32.6% while the CS rate in group 5 was 95.3%. Groups 2 and 4 had higher CS rates due to the significant contribution of the subgroups 2b and 4b (nulliparous and multiparous women, single cephalic at term who underwent CS section before labour started). About 70% if the breeches (groups 6 and 7) had a CS. In group 10, delivery was by CS in 50.5% of the cases. Group 3 was the most important contributor to the total number of CS with 25.3%, followed by group 1 with 24.2% and group 5 with 16.5%.

**Table 2:** The Robson reporting table and neonatal outcomes by Robson group, Princess Christian Maternity Hospital, Sierra Leone, October 2020 to January 2021 (n=1998)

Group*	Of women in the group	Number of CS in the group	Group size (%)	Group CS rate (%)	Absolute group contribution to overall CS rate (%)	Relative contribution of group to overall CS rate (%)
1	559	238	28.0	42.6	11.9	24.0
2	71	58	3.6	81.7	2.9	5.9
2a	19	6	1.0	31.6	0.3	0.6
2b	52	52	2.6	100	2.6	5.2
3	763	249	38.2	32.6	12.5	25.1
4	80	63	4.0	78.8	3.2	6.4
4a	24	*7*	1.2	29.2	0.4	0.7
4b	56	56	2.8	100	2.8	5.6
5	171	163	8.6	95.3	8.2	16.4
6	46	33	2.3	71.7	1.7	3.3
7	58	40	2.9	69.0	2.0	4.0
8	99	66	4.9	66.7	3.3	6.7
9	21	20	1.0	95.2	1.0	2.0
10	107	54	5.4	50.5	2.7	5.4
Unclassifiable	23	8	1.1	34.8	0.4	0.8
**Total**	**1998**	**992**	**100**	**49.6**	**49.6**	**100**

CS. ___________________________________________________________________________________________________________Group size (%)=n of women in the group / total N women delivered in the hospital x ×100.

Group CS rate (%)=n of CS in the group / total N of women in the group x ×100.

Absolute contribution (%)=n of CS in the group / total N of women delivered in the hospital x ×100.

Relative contribution (%)=n of CS in the group / total N of CS in the hospital× x 100.

* Birth weight ≥2500 g was used as a proxy for gestational age >37 weeks.

CScaesarean section

There were nine cases of maternal death in the study population with an overall maternal mortality rate of 0.5%. Severe neonatal outcomes accounted for: 147 (7.4%) stillbirths and eight early neonatal deaths (0.3%) with a perinatal mortality of 7.8%. Of the 1820 live births, 293 (16.1%) reported an Apgar score <7 at 5 min. The outcomes for mothers and newborns, categorised by Robson classes, are provided in the online supplemental material.

**Figure 1:**
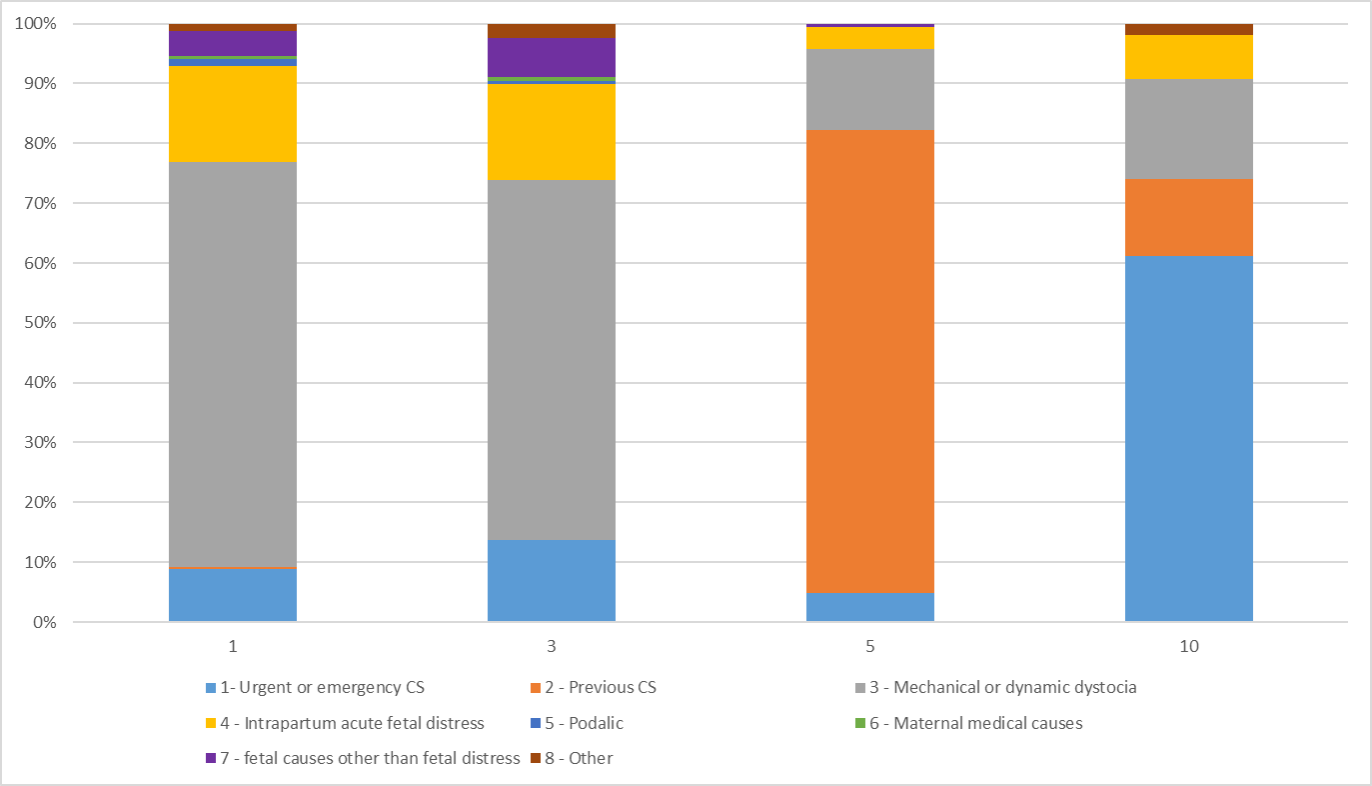
CS Indication among representative Robson groups, 1 October 2020 to 31 January 2021, Princess Christian Maternity Hospital, Sierra Leone (n.984). CS, caesarean section.

Overall, the most frequent indication for CS was dystocia with 384 (39.0%) cases, followed by urgent or emergency indications (163; 16.6%), previous CS (141; 14.3%) and intrapartum acute fetal distress (128; 13.0%). More than half of the emergencies were related to severe pre-eclampsia and eclampsia (92/163; 56.4%). Figure 1 shows the distribution of CS indications in specific Robson groups: low-risk groups 1 and 3, group 5 as women with a previous CS, group 10 as women with preterm births.

In groups 1 and 3, the major indication for CS was dystocia followed by intrapartum acute fetal distress. In group 5, about 80% of the CS had previous CS as the indication, while in high-risk group 10, emergencies were the most frequently reported indications followed by dystocia.

## Discussion

The Robson classification has proven to be easily applicable even in settings with high organisational complexity and a large number of births, such as PCMH. In fact, only 1.2% of the records of women who gave birth during the study period lacked the necessary information to allow its application. However, the need for volunteer intervention for dedicated data collection indicates that, for now, such evaluations are only being captured for research purposes and the conditions are not yet in place for them to be performed routinely and continuously as recommended.^29 30^ During the study period, the overall CS rate at the PCMH was 49.6%, consistent with the rapid increase previously observed from 29.6% in 2016 to 47.6% in 2020.^20^ The maternal and perinatal mortality in the study were, respectively, 0.5% (9 maternal deaths) and 7.8% (148 stillbirths and 8 early neonatal death). Robson groups 1 and 3 were the largest (two-thirds of the women giving birth in PCMH) showing that most women enter labour spontaneously. Robson groups 1, 3 and 5 cumulatively contributed more than 65% of the total of CS (24%, 25.1% and 16.4%, respectively), consistent with other analysis reported from Africa.^23 26 31^ The CS rates in group 1 and 3 were very high, 42.6% and 32.6%, respectively,^17^ with poor neonatal outcomes, with 4.1% of perinatal mortality and 15% of newborns with Apgar scores below 7 at 5 min in group 1, and 5.8% of perinatal mortality and 13.2% of newborns with Apgar scores below 7 at 5 min in group 3. In-depth analysis of these groups are warranted.

The WHO Robson Implementation Manual suggests that the ratio between spontaneous and induced women (group 1: group 2) should be 2:1 or higher. In PCMH, the ratio is 7:1 which is extremely high and may suggest that an increase in the rate of inductions is expected to be beneficial. The overall rate of births with induction in our population was 3.1%. Although it is a value similar to the average of 4.4% observed in other African countries,^32^ it may be insufficient considering the suboptimal outcomes. As in many low-resource settings, many factors hinder the appropriate use of induction of labour in PMCH, such as delayed access of pregnant women to health facilities due to transport difficulties and socioeconomic barriers, scarcity or unavailability of drugs, poor antenatal care attendance, poor training of health personnel and traditional beliefs.^33 34^

As a tertiary level and referral hospital for a large catchment area, PCMH receives many high-risk cases and obstetrical complications. Despite the high CS rate registered at the hospital level, the CS rate at the population level remains very low at less than 5%.^35^ In many low-resourced setting, the referral status and therefore the emergencies received, contribute substantially to the CS rate.^36^ In PCMH, referred women accounted for more than 30% of total admissions in 2020,^20^ and are likely to contribute substantially to the high CS rate within the hospital. Because birth registries did not report referral status, we could not report the referral rate among the women who underwent CS in the study.

Dystocia was the indication in 19% of the CS, similar to that found in a multicentre study in sub-Saharan Africa of 18%.^37^ Dystocia was the most frequent indication for CS in groups 1 and 3, responsible for approximately 50% of CS for both groups, similar to what was observed in other studies in Tanzania^23^ and Uganda.^38^ Dystocia is the major indication for primary CS^39^,^41^ especially among nulliparous women^40 41^ and our findings support this evidence (24% CS for dystocia in nulliparous vs 16% CS for dystocia in multiparous in our study). This warrants an in-depth analysis to assess the management of women with dystocia. Improving the access and quality of antenatal care^41 42^ as well as the appropriate utilisation of partograph and intrapartum fetal monitoring are known as crucial strategies to prevent CS in women with dystocia.^43^ However, in PCMH, referrals of complicated deliveries from birth centres without the capacity to perform CS contribute significantly to the dystocia diagnosis.^36^ The dramatic discrepancy between the CS rate in PCMH versus the population-based CS rate is consistent with this observation.^15^ We endorse the previous evidence calling for a nationwide effort aimed at increasing the availability of this life-saving procedure.^15^

In our study, over 70% of CSs were performed by surgical technicians. However, unfortunately, it was not possible to record who made the decision to perform the CS, and consequently, we are unable to investigate the decision-making process underlying the different indications for CS. This aspect warrants further investigation, especially considering that the PCMH has transitioned into a University Teaching Hospital and can now rely on mentors and residents who can guide less skilled personnel. Furthermore, the introduction of a mandatory second opinion for CS indication has been recommended to reduce CS births in settings with adequate resources.^4^ However, considering the low-resource, understaffed healthcare system in Sierra Leone, we recognise that this intervention may be difficult to implement in this context.

The low proportion of operative vaginal deliveries (0.6%) also is likely to contribute to the high number of CS, as also observed in a study conducted in Ethiopia.^44^ This aspect can be addressed by improving staff training in the identification of women suitable for the procedure and in the use of forceps and vacuum extractor.^45^

The CS rate in women with a previous CS (group 5) was 95%. Although guidelines suggest that rates of 50–60% are appropriate, much higher rates are observed particularly in low and middle income countries.^26^ This finding also suggests a great difficulty in attempting a trial of labour after caesarean section (TOLAC). In PCMH, TOLAC was rarely proposed to eligible women which would be subject to the skills of the medical team on duty. The scarcity of equipment for monitoring the woman in labour, the unavailability of cardiotocographic monitoring and the absence of accurate data regarding fetal biometry, make it very difficult to ensure the proper management of TOLAC on eligible women, as other studies in Africa suggest.^46^,^48^

Robson groups 2 and 4 recorded disproportionate CS values (81.6% and 78.7%, respectively) when compared with the reference values of 25–30% and 15%.^17^ The low number of inductions (groups 2a and 4a; 6 and 7 women, respectively) compared with the higher numbers of pre-labour CS (groups 2b and 4b; 52 and 56 women, respectively) explained the resulting high CS rate in groups 2 and 4. Among the CS in group 2b, 21 (40.3%) were urgent or emergency CS, 17 (32.7%) had indication of intrapartum acute fetal distress. Similarly, in group 4b, 26 (46.4%) had an emergency indication, while 11 (19.6%) were performed for intrapartum fetal distress.

Robson group 10 (premature fetuses) represented about 5% of the women in PCMH and had a CS rate of 50.4%. This high CS rate is usually related to numerous cases of high-risk pregnancies, as fetal growth restriction or eclampsia,^17^ requiring to terminate the pregnancy despite prematurity. Excessive recourse to CS in these cases could be explained by the fear of providers to potential peripartum or intrapartum complications.^11^ In sub-Saharan Africa, the prevalence of hypertensive disorders in pregnancy is high, up to 8%.^49^ At PCMH, 11% of women were diagnosed with hypertensive disorders in 2020, and for 16 women it was the cause of peripartum maternal death, contributing 17% to the total maternal deaths in the year (n=48). In our study, almost one-third of the eclampsia cases occurred in group 10 (^16^; 29.3%), which could explain the high CS rate of the group. On the other hand, dystocia accounted for more than 18% of the CS in group 10, which is counterintuitive to the characteristics of the group itself. This suggests that there may be misreporting of indications, misdiagnosis or misclassification of women (term women classified in this group incorrectly). In-depth analyses of this group, including assessment of gestational age are warranted. The development of protocols for standardised classification of indications reinforcing diagnostic pathways for obstetrical complications should be considered.

Our study has some limitations. The indications for CS were extracted retrospectively from the patients’ medical records at face value and when multiple indications were reported, we used a hierarchy to assign the indication.^24^ The low reproducibility of classifying indications is well-recognised in the literature.^22^ This is exacerbated in PCMH by the absence of guidelines for reporting and classifying CS indications. For twin pregnancies, only outcomes for the first baby were recorded, which may have underestimated the proportion of adverse neonatal outcomes. Maternal and neonatal deaths occurring after discharge were not captured, therefore maternal and perinatal mortality may have been under-reported. In the absence of reliable data on gestational age, we used birth weight as a proxy, an approximation that has been used in earlier studies implemented in low-resource settings. Lastly, data collection took place during the COVID-19 pandemic, which may have impacted hospital access, deliveries, obstetrical complications and CSs. Further studies could be conducted to investigate the role of the pandemic on CS rates in these settings.

## Conclusion

Our study showed that half of the women who give birth at PCMH underwent CS. Analysis using the Robson classification depicts that groups 1 and 3 constitute two-thirds of the obstetrical population and present very high CS rates (43% and 33%, respectively) with poor newborn outcomes despite being usually considered at low risk. While dystocia was the leading indication for CS in these groups (about 60%), induction of labour may be underused, contributing to suboptimal outcomes. Almost all women with a previous CS underwent CS again (95%), showing rare recourse to TOLAC.

The more appropriate use of labour induction, careful monitoring of obstetrical complications and intrapartum maternal-fetal status, effective training to conduct operative deliveries and TOLAC could be key strategies to improve the appropriate use of CS and the quality of obstetrical care. However, the interpretation of the high number of CSs should take into account that PCMH centralises complicated cases from a very wide catchment area, and the population CS rate remains insufficient according to WHO recommendations. The evaluation of CS according to the Robson classification should be routinely and prospectively introduced into clinical practice to improve the quality of the information collected and enable the monitoring of quality improvement interventions. Further research should be carried out to investigate the contribution of cases referred from other facilities to the CS rate at the hospital level, and an in-depth analysis of the management of women with dystocia and with a previous CS.

## supplementary material

10.1136/bmjopen-2023-081143 online supplemental file 1

## Data Availability

Data are available upon reasonable request.
