# Supplementary material for: Caesarean sections, indications and outcomes: a cross-sectional study using the Robson classification in a tertiary hospital in Sierra Leone
Source: BMJ Open. 2024 Sep 3;14(9):e081143. doi: 10.1136/bmjopen-2023-081143 (PMC11409385; doi:10.1136/bmjopen-2023-081143)
Supplement: online supplemental file 1 [file bmjopen-14-9-s001.pdf]

Supplemental Table. The Robson reporting table and neonatal outcomes by Robson group, PCMH Hospital, Sierra Leone, October 2020 – January 2021 (n = 1998)

GROUP*	N. of women in the group	Number of CS in the group	Group size (%)	Group CS rate (%)	Absolute group contribution to overall CS rate (%)	Relative contribution of group to overall CS rate (%)	Maternal Death (N death woman/live births (%))	Livebirth (N livebirths/total deliveries in the group (%))	Fresh Stillbirth (N Fresh stillbirths/total deliveries in the group (%))	Macerated Stillbirth (N Macerated stillbirth/total deliveries in the group (%))	Early Neonatal Death**† (N neonatal deaths/total deliveries (%))	APGAR <7 at 5 minutes (N live birth Apgar <7/total live birth (%))
1	559	238	28.0	42.6	11.9	24.0	1 (0.05)	536 (95.9)	18 (3.2)	2 (0.4)	3 (0.5)	83 (15.5)
2	71	58	3.6	81.7	2.9	5.9	0	59 (83.1)	12 (16.9)	-	-	10 (16.9)
2:00 AM	*19*	*6*	*1.0*	*31.6*	*0.3*	*0.6*	*0*	***13 (68.4)***	*6 (31.6)*	-	-	*3 (23.1)*
2 b	*52*	*52*	*2.6*	*100*	*2.6*	*5.2*	*0*	***46 (88.5)***	*6 (11.5)*	-	-	*7 (15.2)*
3	763	249	38.2	32.6	12.5	25.1	2	718 (94.1)	35 (4.6)	6 (0.8)	4 (0.5)	101 (14.1)
4	80	63	4.0	78.8	3.2	6.4	1 (0.05)	69 (86.3)	10 (12.5)	1 (1.3)	-	17 (24.6)
4:00 AM	*24*	*7*	*1,2*	*29.2*	*0.4*	*0.7*	*0*	*19 (79.2)*	*5 (20.8)*	-	-	*6 (31.6)*
4 b	*56*	*56*	*2.8*	*100*	*2.8*	*5.6*	*1 (0.05)*	*50 (89.3)*	*5 (8.9)*	*1 (1.8)*	-	*11 (22.0)*
5	171	163	8.6	95.3	8.2	16.4	0	165 (96.5)	6 (3.5)	-	-	15 (9.1)
6	46	33	2.3	71.7	1.7	3.3	0	38 (82.6)	7 (15.2)	-	1 (2.2)	14 (36.8)
7	58	40	2.9	69.0	2.0	4.0	0	51 (87.9)	6 (10.3)	1 (1.7)	-	18 (35.3)
8	99	66	4.9	66.7	3.3	6.7	2 (0.1)	93 (93.9)	5 (5.1)	1 (1.0)	-	10 (10.8)
9	21	20	1.0	95.2	1.0	2.0	0	20 (95.2)	1 (4.8)	-	-	3 (15.0)
10	107	54	5.4	50.5	2.7	5.4	3 (0.1)	71 (66.4)	33 (30.8)	3 (2.8)	-	22 (31.0)
Unclassifiable	23	8	1.1	34.8	0.4	0.8	Missing	18 (78.3)	Missing	Missing	Missing	1 (5.6)
**TOT**	**1998**	**992**	**100**	**49.6**	**49.6**	**100**	**9 (0.5)**	**1838 (92.0)**	**133 (6.7)**	**14 (0.7)**	**8 (0.4)**	**294 (16.0)**

*Birth weight ≥2500 g was used as proxy for gestational age >37 weeks
**Early neonatal death was defined as the death of a live born neonate by discharge or day 7 of life (whichever occurred first)
CS. caesarean section
Group size (%) = n of women in the group / total N women delivered in the hospital x 100
Group CS rate (%) = n of CS in the group / total N of women in the group x 100
Absolute contribution (%) = n of CS in the group / total N of women delivered in the hospital x 100
Relative contribution (%) = n of CS in the group / total N of CS in the hospital x 100
