# Supplementary material for: Caesarean sections, indications and outcomes: a cross-sectional study using the Robson classification in a tertiary hospital in Sierra Leone
Source: BMJ Open. 2024 Sep 3;14(9):e081143. doi: 10.1136/bmjopen-2023-081143 (PMC11409385; doi:10.1136/bmjopen-2023-081143)
Supplement: Supplementary file 2 [file bmjopen-2023-081143.reviewer_comments.pdf]

PEER REVIEW HISTORY

BMJ Open publishes all reviews undertaken for accepted manuscripts. Reviewers are asked to complete a checklist review form and are provided with free text boxes to elaborate on their assessment. These free text comments are reproduced below.

ARTICLE DETAILS

**TITLE (PROVISIONAL)**	Caesarean sections, indications and outcomes: a cross sectional study using the Robson classification in a tertiary hospital in Sierra Leone
**AUTHORS**	Arata, Matteo; Boyle, Sonia; Sgorbissa, Beatrice; Tognon, Francesca; John-Cole, Valerie; Michele, Orsi; Claudia, Caracciolo; Saccardi, Carlo; Manenti, Fabio; Putoto, G; K. Kamara, Abibatu; Betran, Ana Pilar

VERSION 1 - REVIEW

**REVIEWER NAME**	Adama, Ouattara
**REVIEWER AFFILIATION**	University of Ouagadougou
**REVIEWER CONFLICT OF INTEREST**	No competing interest.
**DATE REVIEW RETURNED**	08-Nov-2023

**GENERAL COMMENTS**	At the abstract level, the author should revise the structure of the abstract to reflect certain important methodological aspects.<br><br>At the methodological level, the author should calculate the minimum sample size to be studied on the basis of data in the literature.

**REVIEWER NAME**	Tontus, H Omer
**REVIEWER AFFILIATION**	Istanbul Technical University, Molecular Biology & Genetics
**REVIEWER CONFLICT OF INTEREST**	free from competing interests.
**DATE REVIEW RETURNED**	29-Nov-2023

**GENERAL COMMENTS**	Although it was stated that ethical approval had been granted, a registration number for approval documents was not provided.

**REVIEWER NAME**	Jauniaux, Eric
**REVIEWER AFFILIATION**	UCL, EGA Institute for Women Health
**REVIEWER CONFLICT OF INTEREST**	No conflict of interest.
**DATE REVIEW RETURNED**	08-Feb-2024

**GENERAL COMMENTS**	The authors present their data on the use of the Robson's 'Ten Groups Classification' to evaluate the CS indications and rates in a tertiary hospital in Freetown, Sierra Leone capital city. They report an unusually high rate of CS (almost 50%, these are rates found Latin America, South of Italy, Greece, Egypt, Turkey) for a sub-

	Saharan country (these are North African and Latin America rates) with dystocia the leading indication for CS (2/3) in groups 1 and 3 and overall poor perinatal outcomes. They conclude that use of labour induction, appropriate diagnosis and management of obstetrical complications, second opinion for CS indication, training to conduct operative deliveries and recourse to trial of labour after CS could be key strategies to improve the appropriate use of CS.<br><br>This is a retrospective study with data obtained over 4 months at the peak of the covid pandemic which in itself could have had an impact on the data analysis. Having organized numerous training obstetrics programs on basic imaging, management of obstetric and neonatal labor complications and cesarean deliveries in many different LMICs countries over the last 15 years, I strongly disagree with the author' statement that their data can be compared with those of similar settings due to major variations between and within countries (and continents) in maternity health care provision in terms which the authors highlight themselves in their introduction and material and methods.<br>- In many countries, there are very few trained obstetricians-gynaecologists are they have either left spontaneously left the country or been poached by Western countries and thus most cesarean deliveries are performed by GPs or medical officers and those are mainly based in capital cities. The authors should stratify their data according to the level of training/expertise of the health care providers.<br>- There are also wide variations between capital cities regarding patient charges (with an incentive to do CS as the doctors are paid more for those) and distribution of health care with private clinics having mushroomed in many capital cities with draining of local resources in term of medical drugs, transfusion, a surgical expertise but also imaging and fetal monitoring equipment. The authors should detail the availability of these resources in their setting and explain how this could be associated with such a high rate of CS in their maternity (1-2 fold higher that CS rates in private clinics in other sub-Saharan African countries).<br>- Most deliveries in sub-Saharan African countries take place in rural area and the patients. Their suggestions on second opinion for CS indication and trial of labor/scar contrast strongly with the lack of support for local major basic issues in providing basic obstetric care, unrealistic and influenced by a Western view of the rest of world. Within this context, the Robson classification may not be ideal for low-resource set-ups and the authors should be more nuanced in their discussion regarding its use in LICs. Perhaps this should be the focus of their discussion and compare their results with those of Western countries and in particular Italy where the rates of CS are now > 50% in the south of the country.

**REVIEWER NAME**	Jayawardane, Asanka
**REVIEWER AFFILIATION**	University of Colombo Faculty of Medicine, Obstetrics and Gynecology
**REVIEWER CONFLICT OF INTEREST**	None
**DATE REVIEW RETURNED**	15-Feb-2024

**GENERAL COMMENTS**	Congratulations to the authors who have studied a very important issue in an extremely challenging set up. The study benefits from the use of ten group classification- which allows objective analysis of

	the CS rates- also making the results generalisable and comparable. Also the authors have successfully described the specific challenges and limitations specific to their setting.<br>The study setting seems extremely challenging- however authors (by using 10 group classification) have managed to comparatively analyse the situation with similar settings published in the literature- a key strength of the ten group classification. It will be a tremendous improvement to the current discussion if the authors can include a deeper comparative analysis of the cited literature with their own results.<br><br>1. One of the most important findings of the study is the absence of key data- which should be highlighted with the population specific solutions looked into.<br><br>2. The study highlights another key issue- very high CS rates for term spontaneous labour.<br>With significant mortality rates AND near universal repeat CS rates, preventing the first/ primary CS takes a supreme importance in saving lives and I would urge the authors to explore more into the results of the groups 1 and 3. If necessary, this should be planned as a new study and results combined for a very interesting publication! Without deeper exploration the study results describe a single step in an interesting audit cycle.<br><br>3. It is not clear why the authors conclude that increased rates of induction of labour will likely reduce the CS rates in groups 1 and 3.<br><br>4. Suggest that the discussion also guide/ include any known/ reported interventions that may reduce the CS rates and/or improves outcomes in similar and comparable study settings and the challenges PCMH may face in implementing those.

**REVIEWER NAME**	***Bakker, Wouter***
**REVIEWER AFFILIATION**	St. Luke's Hospital, Clinical Department
**REVIEWER CONFLICT OF INTEREST**	None
**DATE REVIEW RETURNED**	06-Mar-2024

**GENERAL COMMENTS**	Dear authors,<br><br>Thank you for this manuscript and important insights in hospital statistics. I can imagine this can help a lot with addressing needs in the facility but also other similar facilities.<br><br>I have a few remarks on the manuscript.<br><br>Abstract<br>Line 50: I find extreme emergencies here somewhat vague, please use a different term or elaborate<br><br>Introduction<br>Line 93: anesthesia complications-related. I assume you mean anaesthesia-related complications?<br>Line 94: could you elaborate on the risk of incontinence and pelvic organ prolapse as complications of caesarean section?<br>Line 97: I would rephrase poor management, maybe into suboptimal management?

	Methods<br>Line 170: does pre-eclampsia fall under extreme emergencies? Usually there is time to properly plan CS or induce labour? Same with placenta praevia (without blood loss), this is more often an elective caesarean section indication<br>Line 172: would cord prolapse not fall under the extreme emergency caesarean sections?<br><br>Results<br>Table 1: was the CS operator also the indicator? This would maybe even be of more importance, since many training programs focus on delivering skills (performing the procedure) but sometimes lack the teaching of proper diagnosing and decision-making<br>Line 217/218: I would for readibility explain briefly which women these groups entail.<br><br>Discussion<br>Line 272: what guidelines suggest this ratio?<br>Line 288: how was dystocia defined?<br>Line 329: I would not consider this scope of this study so would omit this part (calcium supplementation)<br><br>What I generally miss in the discussion is elaboration on the shockingly high caesarean section rates for especially multigravida without caesarean section, and on the high volume of dystocia indications. Hereby is proper diagnosis of prolonged labour / dystocia crucial. I advise to provide more background as to how this was done in the hospital. How was labour progress monitored, who made indications, what could be reasons that caesarean section rates are this high. This will provide opportunities for further research (which you highlighted) and stresses the urgency of tackling these caesarean section rates.

VERSION 1 – AUTHOR RESPONSE

Reviewer: 1
Dr. Ouattara Adama, University of Ouagadougou
Comments to the Author:
At the abstract level, the author should revise the structure of the abstract to reflect certain important methodological aspects.

Thank you very much for this feedback. The abstract structure adheres to the journal guidelines, but we have now incorporated an additional paragraph to provide a more comprehensive description of the outcomes.

Primary and secondary outcome measures: Primary outcome: CS rate by Robson Group. Secondary outcomes: indications for CS and the newborn outcomes for each Robson Group.

At the methodological level, the author should calculate the minimum sample size to be studied on the basis of data in the literature.

Thank you for your suggestion. In literature, the sampling for evaluating the CS rate according to Robson is highly diverse. For accurate evaluation and comparison, ideally, annual data for multiple

years would be preferable for comparison, as suggested by the platform provided by WHO (https://robson-classification-platform.srhr.org/).

However, the sample size greatly depends on the ability to gather information, which is particularly relevant in studies conducted in developing countries. For instance, a recent study conducted at a tertiary hospital in Nigeria collected data from 447 women between August 2020 and February 2022 (Akadri, A.A., Imaralu, J.O., Salami, O.F. et al. Robson classification of caesarean births: implications for reducing caesarean section rate in a private tertiary hospital in Nigeria. BMC Pregnancy Childbirth 23, 243 (2023). https://doi.org/10.1186/s12884-023-05557-x).

In our study, limited resources constrained the data collection to four months. However, during this four months, ALL women giving birth in the hospital were included so no selection bias were introduced. During these months, the presence of one resident doctor (MA) and one Health Officer (SB) who dedicated themselves prospectively to collecting the necessary data for classification made this analysis possible. A retrospective data collection based on paper records would not have been feasible to increase the sample size because some information, such as fetal position and details on previous deliveries, were not systematically and routinely collected and recorded.
We have now added in the Variables, data collection, and analysis paragraph, "The presence of two dedicated volunteer doctors facilitated data collection during the study period," to better explain the rationale for evaluating this particular sample.

Reviewer: 2
Dr. H Omer Tontus, Istanbul Technical University
Comments to the Author:
Although it was stated that ethical approval had been granted, a registration number for approval documents was not provided.

Dear Reviewer, thank you for highlighting this aspect.

Unfortunately, we do not have a registration number as it was not provided to us by the competent body for national ethical approval. However, we have now added the date when we obtained approval for both retrospective and prospective data collection of the variables necessary for the study.

Permission to perform the study was obtained from the hospital management team and ethical approval was obtained from Sierra Leone Ethics and Scientific Review Committee on December 16th, 2020.

Reviewer: 3
Prof. Eric Jauniaux, UCL
Comments to the Author:

The authors present their data on the use of the Robson's 'Ten Groups Classification' to evaluate the CS indications and rates in a tertiary hospital in Freetown, Sierra Leone capital city. They report an unusually high rate of CS (almost 50%, these are rates found Latin America, South of Italy, Greece, Egypt, Turkey) for a sub-Saharan country (these are North African and Latin America rates) with dystocia the leading indication for CS (2/3) in groups 1 and 3 and overall poor perinatal outcomes.

They conclude that use of labour induction, appropriate diagnosis and management of obstetrical complications, second opinion for CS indication, training to conduct operative deliveries and recourse to trial of labour after CS could be key strategies to improve the appropriate use of CS.

This is a retrospective study with data obtained over 4 months at the peak of the covid pandemic which in itself could have had an impact on the data analysis.

Having organized numerous training obstetrics programs on basic imaging, management of obstetric and neonatal labor complications and cesarean deliveries in many different LMICs countries over the

last 15 years, I strongly disagree with the author' statement that their data can be compared with those of similar settings due to major variations between and within countries (and continents) in maternity health care provision in terms which the authors highlight themselves in their introduction and material and methods.

Dear reviewer, we really thank you for your comment and clarification, which allows us to better explain the context in which we conducted the study.

First and foremost, We agree that each maternity is different and influenced by specific circumstances and factors that shape practices and interventions, including the consideration and use of cesarean section.

We are aware that the data collection conducted during the Covid-19 pandemic may have influenced the findings of this study as comprared with data before covid pandemic. We had mentioned this among the limitations at the end of the Discussion section ("Lastly, data collection took place during the Covid19 pandemic, which may have impacted hospital access, deliveries, obstetric complications, and caesarean sections. Further studies could be conducted to investigate the role of the pandemic on CS rates in this settings.").

As described in previous studies, the pandemic has resulted in reduction of hospital admissions, although maternal and pediatric care services have experienced a lesser decline compared to adult outpatient or surgical services. (Sevalie S, Youkee D, van Duinen AJ, et al. The impact of the COVID-19 pandemic on hospital utilisation in Sierra Leone. BMJ Global Health 2021;6:e005988. doi:10.1136/bmjgh-2021-005988; Quaglio G, Cavallin F, Nsubuga JB, Lochoro P, Maziku D, Tsegaye A, Azzimonti G, Kamunga AM, Manenti F, Putoto G. The impact of the COVID-19 pandemic on health service use in sub-Saharan Africa. Public Health Action. 2022 Mar 21;12(1):34-39. doi: 10.5588/pha.21.0073. PMID: 35317534; PMCID: PMC8908870.).

An increase in the CS rate during that period was certainly influenced by the fact that emergency management and referral services did not experience a decline in activities. Consequently, it is possible that a higher percentage of severe cases and complications reached the referral hospital, at the expense of uncomplicated births that could have been managed by peripheral facilities with lower levels of care intensity.

Although the data is not available to prepare the Robson classification in previous years, routine hospital data collection shows that the CS rate in PCMH has shown a gradual increase over time from less than 30% until 2018 to approximately 43% by 2022. See Figures below.

The aim of the study was to capture the phenomenon of the gradual increase in the CS rate in a more meaningful manner using the Robson Classification (since the overall CS rate is a very limited metric for maternity services) which could serve as a baseline to analyze the trend and the effect of any interventions that may be implemented in the future aiming at improving the quality of care.

We are aware that the context must be taken into consideration, and it can be challenging to compare the reality of the PCMH with other similar contexts due to the uniqueness of the hospital (being a referral and teaching hospital, exclusively obstetric/gynecological, with over 6000 deliveries per year). Nevertheless, we believe that a tool like the Robson classification can be useful if applied consistently and, most importantly, evaluated alongside other variables including events and processes. For this reason, we did not limit ourselves to describing the cesarean section rates within the Robson classes. Instead, we expanded data collection to important outcomes (e.g. stillbirths, maternal and neonatal deaths, as well as indications for caesareans) in each Robson group as done in other contexts (Tognon F, Borghero A, Putoto G, et al. Analysis of caesarean section and neonatal outcome using the Robson classification in a rural district hospital in Tanzania: an observational retrospective study. BMJ Open

2019;9:e033348. doi: 10.1136/bmjopen-2019-033348), highlighting how being a referral hospital and managing numerous emergencies could influence this cesarean section rate. This is increasingly being reported in the literature (e.g. Sørbye IK, Vangen S, Oneko O, Sundby J, Bergsjø P. Caesarean section among referred and self-referred birthing women: a cohort study from a tertiary hospital, northeastern Tanzania. BMC Pregnancy Childbirth. 2011 Jul 28;11(1):55).

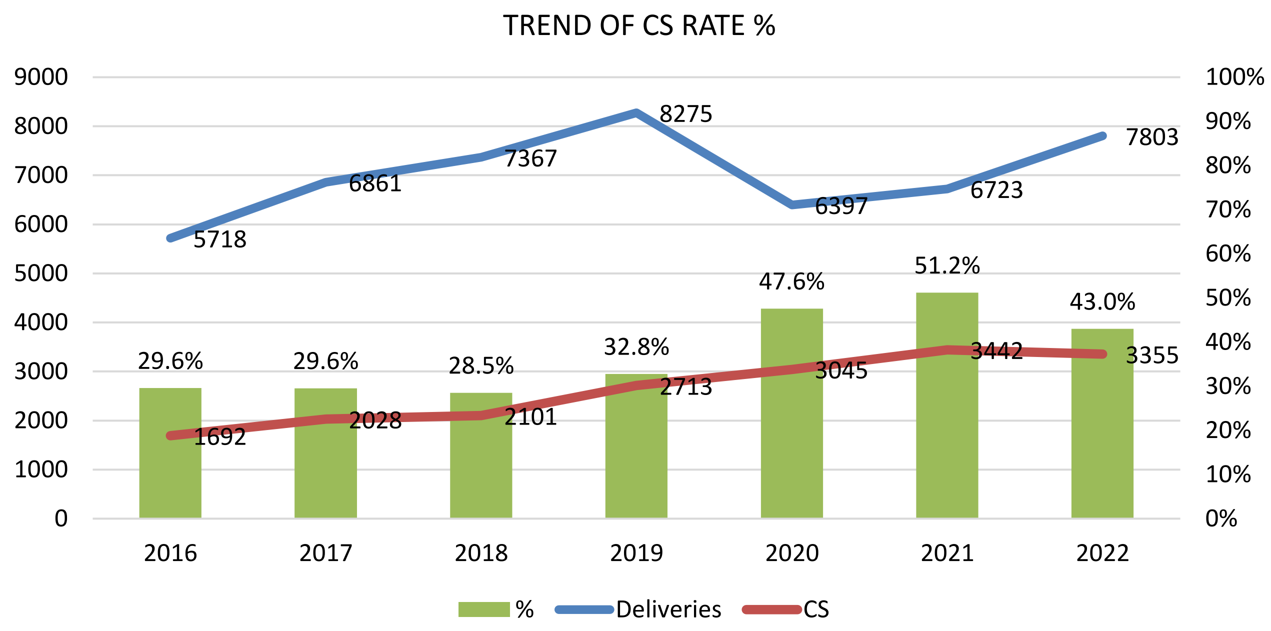

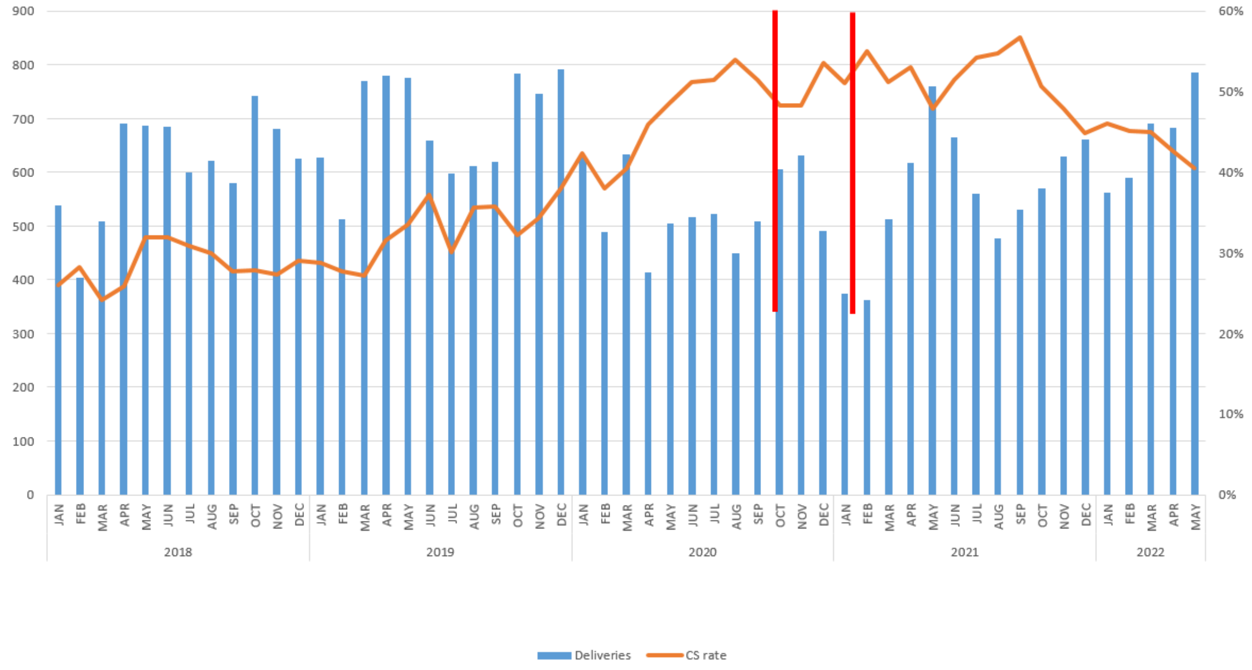

- In many countries, there are very few trained obstetricians-gynaecologists are they have either left spontaneously left the country or been poached by Western countries and thus most cesarean deliveries are performed by GPs or medical officers and those are mainly based in capital cities. The authors should stratify their data according to the level of training/expertise of the health care providers.

Thank you very much for emphasizing this important aspect. Both type of provider in charge of the decision and in charge of performing a cesarean section are important dimension for a more in-depth understanding of the decision-making process and potential adherence to guidelines. Unfortunately, we were unable to retrieve information on which healthcare providers made the decision to perform CS, only data on who performed it.

When comparing the percentages of cesarean sections performed for each Robson group, we did not observe any significant differences between specialists and technical officers (see Figure below). Therefore, we did not further dwell on this aspect in the result description.

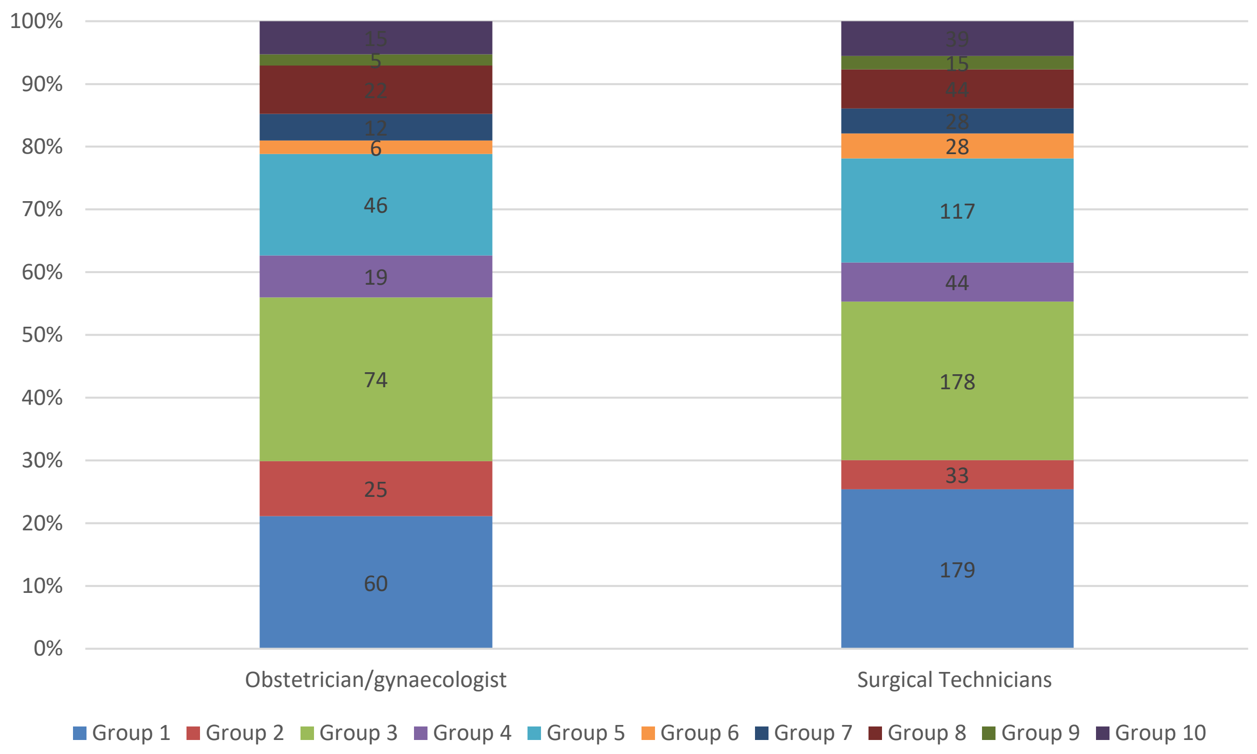

However, as you suggested, we have further emphasized in the discussion the importance of considering this aspect in future studies:

In our study, over 70% of CSs were performed by surgical technicians. However, unfortunately, it was not possible to record who made the decision to perform the CS, and consequently, we are unable to investigate the decision-making process underlying the different indications for CS. This aspect likely warrants further investigation, especially considering that the PCMH has transitioned into a University Teaching Hospital and can now rely on mentors and residents who can guide less skilled personnel.

- There are also wide variations between capital cities regarding patient charges (with an incentive to do CS as the doctors are paid more for those) and distribution of health care with private clinics having mushroomed in many capital cities with draining of local resources in term of medical drugs, transfusion, a surgical expertise but also imaging and fetal monitoring equipment. The authors should detail the availability of these resources in their setting and explain how this could be associated with such a high rate of CS in their maternity (1-2 fold higher that CS rates in private clinics in other sub-Saharan African countries).

Thank you for this reflection. Private intra-hospital activity in obstetric care is minimal in this context, accounting for less than 3% of procedures in the PCMH activity. Private clinics in the city rely on PCMH as referral centre in the same way as public peripheral health centres which cannot perform caesarean sections, so they refer patients with complications to the government hospital.

In the context of PCMH, the component related to gynecological care is privatized, while obstetric care should benefit from Free Health Care as per government policy.

We agree that the phenomenon of out-of-pocket payments directly to the surgeon may be present in this context, but it is a phenomenon that is difficult to quantify and it rarely involve the part dedicated to the management of obstetric complications, which is the focus of this study.

In addition, as we have explained above and as per the Figure above, we think that the high CS rate is the consequence of a covid pandemic period where cases with severe complications reached the hospital but women with uncomplicated births stayed and gave birth at home resulting in a higher risk obstetric population. Unfortunately, we lack previous data using the Robson classification for a trend analysis.

- Most deliveries in sub-Saharan African countries take place in rural area and the patients. Their suggestions on second opinion for CS indication and trial of labor/scar contrast strongly with the lack of support for local major basic issues in providing basic obstetric care, unrealistic and influenced by a Western view of the rest of world.

Thank you for emphasizing this important point. We agree that in the majority of sub-Saharan district and rural hospitals, the consideration of seeking a second opinion could not even be entertained due to the lack of personnel. However, in the context of PCMH being a referral hospital and having medical specialists on staff, implementing this procedure may be feasible. Specifically, in our context, if the decision for a surgical delivery has been made by a surgical health technician, we suggest consulting a doctor if available. We believe that in the absence of other restrictions, this could improve decision-making.

This aspect is even more relevant in recent years, following the conduct of our study, as PCMH has transitioned into a University Teaching Hospital as mentioned above. This has consequently increased the availability of personnel capable of serving as mentors and guiding less prepared personnel in labor management and monitoring.

However, we agree with the reviewer that introducing a second opinion in such a challenging context could be very complicated and may not be useful. In fact, WHO recommendation on 2nd opinion is a “context-specific recommendation”. We have added in the discussion the following sentence in order to highlight this consideration:

Furthermore, the introduction of a mandatory second opinion for CS indication has been recommended to reduce CS births in settings with adequate resources (4). However, considering the low-resource, understaffed healthcare system, we recognize that this aspect may be difficult to implement in this context.

Within this context, the Robson classification may not be ideal for low-resource set-ups and the authors should be more nuanced in their discussion regarding its use in LICs. Perhaps this should be the focus of their discussion and compare their results with those of Western countries and in particular Italy where the rates of CS are now > 50% in the south of the country.

We agree that the Robson classification does not cover all the dimensions of labour and childbirh and thus does not provide all the information that would be necessary for an in-depth understanding of the use of caesarean section and practices. The Robson classification does not explain why caesarean sections are done but it does allow an objective, common starting point to investigate the indications among more uniform and comparable groups of women. This is the case for all contries whether high-

or low-income and in all settings whether rich or poor. For this reason, the Robson Classificaiton is consider not an end-point but rather a starting point, a common scheleton within which further practices, outcomes or events can be explored and analyzed. Changes in any perinatal outcomes as a result of altering clinical practices can be monitored easily and more meaningfully using the classification. As the Robson classification is more robust and uses a more “universal language” than other classifications, more reliable comparisons are feasible compared with other classifications in the literature.

However, we also undersand that the is not a panacea and implementing the Robson classification alone will not solve the challenge, Adequate interpretation of the data thtrough the Classification must lead to changes in practice by identifying, analysing and focusing interventions on specific groups of women of particular relevance for each health care facility (e.g. women with specific poor outcomes or women with specific obstetric conditions).It is clear that any changes in practices and clinical protocols deem necessary to improve management must come from the healthcare professionals in the maternity; it requires behavioural changes beyond the initial preparation of the classification table and commitment to the changes and to the decisions taken by the maternity team. In addition, deliverate efforts to regularly use the Robson classification are needed for long-term results and sustained improvement of outcomes.

We respectfully disagree with the reviewer's comment that the Robson classification is not useful in low-resource settings. In fact, the classification has been particularly well appreciated in low-resource setting and numerous publications from this countries corroborate it.

Many of these countries have reported the main strengths of this classification: its simplicity, robustness, reliability and flexibility are particularly useful and relevant in low-resource settings. The classification only requires the use of a limited number of variables. Data collection can be as simple as going manually through each patient record looking for the core variables without the need to high-technology out of the reach of the low-resource settings. It does not either require advance statistical knowledge since very simple calculations are used.

We think that comparisons with Italy or any other high-income countries is not appropriate for the objectives of this manuscript. Context and factors affecting care as well as the care itself are dramatically different. As suggested by the implementation manual, comparisons need to be within the same type of unit (between units or between populations), which is the aspect we focused on during the discussion, comparing PCMH with other referral centers in sub-Saharan Africa. This is an area we aim to further explore in the future, with an evaluation planned in the coming months to compare with the data presented in this article, which can serve as our baseline.

We are very grateful for bringing to our attention that our paper conveyed a perspective of criticism toward the high number of cesarean sections and appeared to lack consideration of the context. We hope that in this revised version, we have been able to provide a more accurate portrayal of our intention, with the awareness that this is just the first step in evaluating a phenomenon that we aim to continue studying and exploring further.

Reviewer: 4
Dr. Asanka Jayawardane, University of Colombo Faculty of Medicine
Comments to the Author:
Congratulations to the authors who have studied a very important issue in an extremely challenging set up. The study benefits from the use of ten group classification- which allows objective analysis of the CS rates- also making the results generalisable and comparable. Also the authors have successfully described the specific challenges and limitations specific to their setting.
The study setting seems extremely challenging- however authors (by using 10 group classification) have managed to comparatively analyse the situation with similar settings published in the literature- a key strength of the ten group classification. It will be a tremendous improvement to the current discussion if the authors can include a deeper comparative analysis of the cited literature with their own results.

1. One of the most important findings of the study is the absence of key data- which should be highlighted with the population specific solutions looked into.

Thank you for your considerations. We fully agree that the lack and difficulty in accessing essential data are unfortunate aspects that greatly hinder the continuous replicability of this assessment. We specified in the methods section that data collection was made possible thanks to the work of two doctors who dedicated themselves to this during their internship. However, the complex organization of a tertiary-level hospital, combined with the lack of electronic records, prevented us from obtaining information that would have been very useful for the evaluation (such as whether the patient was referred from a peripheral healthcare facility or not). The lack of reliable and comprehensive data, and of feasible data-collection mechanisms is a chonic challenge in low-resource settings and hinders in many cases, the implementation of data-driven changes and quality improvement strategies in this settings resulting in a vicious circle. We are committed to leverage and advocate for the need of routine data-collection and analysis with the MoH.

2. The study highlights another key issue- very high CS rates for term spontaneous labour. With significant mortality rates AND near universal repeat CS rates, preventing the first/ primary CS takes a supreme importance in saving lives and I would urge the authors to explore more into the results of the groups 1 and 3. If necessary, this should be planned as a new study and results combined for a very interesting publication! Without deeper exploration the study results describe a single step in an interesting audit cycle.

We absolutely agree that future in-depth studies should focus on classes 1 and 3; for this reason, we dedicated graphical representation in Figure 1 to these classes. Two major analyses have been prompted by the analysis reported in this paper related to groups 1 and 3:

- Since about 70% of the CS in these groups are for mechanical or dinamic distocia, an assessment of the definitions and protocols related to dystocia is warranted. This will result in a deeper understanding of these women and their curernt clinical management as well as possible improvements.
- Understading emergencies that lead to CS in this group. For example PE/E and how these are managed.

The in-dept analysis of the above could certainly lead to changes in management to improve care and outcomes.

Precisely this is the purpose of the Robson classification: to identify the groups of women which contribute most and least to overall CS rates and further conduct in-depth analysis of the reasons, including management, practices or characteristics and deciding on interventions focused on these specific groups of particular need.

We consider this study as the first step in describing a phenomenon that we aim to continue analyzing. These data can serve as a baseline for hospital management to assess how potential organizational changes may affect cesarean section rates within each class.

3. It is not clear why the authors conclude that increased rates of induction of labour will likely reduce the CS rates in groups 1 and 3.

Thank you for highlighting this aspect. The fact that induction is not frequently practiced compared to the reference population is evidenced by the high ratio between class 1 and 2 (7:1). International comparison to data suggest that this ratio is usually 2:1 or higher. A lower ratio may indicate a high induction/prelabour CS issue which may indicate a high risk population in nulliparous women and are likely therefore to have a high CS rate. On the contrary, a very high ratio like in our case, may indicate insuficient induction suggesting to further study pre-labour stillbirth rate which may be elevated if more inductions would save more babies. Alternatively, we may be facing a very low risk population. (See WHO Robson Classification Implementation Manual: https://iris.who.int/bitstream/handle/10665/259512/9789241513197-eng.pdf?sequence=1)

Furthermore, a more careful management of induction could be helpful for some cases of dystocia diagnosis, which we presume may not have been properly monitored.

4. Suggest that the discussion also guide/ include any known/ reported interventions that may reduce the CS rates and/or improves outcomes in similar and comparable study settings and the challenges PCMH may face in implementing those.

Thank you very much for this suggestion. We have now focused and organized the possible actions to be taken in the conclusion section.

The more appropriate use of labour induction, careful monitoring of obstetrical complications and intrapartum maternal-fetal status, effective training to conduct operative deliveries and TOLAC could be key strategies to improve the appropriate use of CS and the quality of obstetric care. However, the interpretation of the high CS rate in groups 1 and 3 need to take into account that PCMH centralizes complicated cases from a very wide catchment area, and that women in these groups may be of higher risk than expected (e.g. women with hypertensive disorders of pregnancy, antepartum haemorrhage). Further research should be carried out to investigate the contribution of cases referred from other facilities to the CS rate at the hospital level, and an in-depth analysis of the management of women with dystocia and with a previous CS.

However, as highlighted to another reviewer, it was not our intention to focus attention specifically on the need to decrease the rate of cesarean sections, but rather to provide information for reflection on the appropriateness of cesarean sections in light of the number of obstetric complication cases that a tertiary-level hospital faces. Even high rates of caesarean section may be justified if the population medically requires it and requires in-depth undersanding of the situation and the obstetric population managed during the time of data-collection.

Reviewer: 5
Dr. Wouter Bakker, St. Luke's Hospital
Comments to the Author:
Dear authors,

Thank you for this manuscript and important insights in hospital statistics. I can imagine this can help a lot with addressing needs in the facility but also other similar facilities.

I have a few remarks on the manuscript.

Abstract
Line 50: I find extreme emergencies here somewhat vague, please use a different term or elaborate

Dear Reviewer, thank you very much for your comment that is giving us the opportunity to revise in a more comprehensive manner the description of the indication for CS.

One of the strengthes of our analysis is the nesting of an indication-based classification within the women-based classification (Robson). For this purpose, we decided to use the classification proposed by Althabe et al in 2004, since this classification scored highest in this category in the the WHO systematic review of classifications (Torloni MR, Betran AP, Souza JP, Widmer M, Allen T, Gulmezoglu M, Merialdi M. Classifications for cesarean section: a systematic review. PLoS One. 2011 Jan 20;6(1):e14566. doi: 10.1371/journal.pone.0014566).

As reported in the Table 4 of the cited paper (https://doi.org/10.1371/journal.pone.0014566.t004), Althabe classification has 8 main cathegories: Extreme emegency, previous CS, dystocia, intrapartum acute feral distress, podalic presentation, maternal causes, fetal causes, Other.

Nevertheless we completely agree that the definition of “extreme emergency” is not well defined and not commonly recognized in the clinical pratice. Therefore, taking into consideration your suggestion and wanting to adhere to the Althabe indication, we decided to reformulate the indication in the following way:

(1) Urgent or emergency CS (considering severe hypertensive disorder including severe pre eclampsia, eclampsia, antepartum haemorrhage due to abruptio placentae or placenta previa, laparatomy for uterine rupture),

(2) previous CS,

(3) mechanical or dynamic dystocia (obstructed and prolonged labour, cephalo-pelvic disproportion, transverse lie, failed induction)

(4) intrapartum acute fetal distress (including cord prolapse)

(5) breech presentation,

6) maternal medical causes (severe anaemia, sickle cell disease, severe malaria)

(7) fetal causes other than fetal distress (macrosomia, idrocefalo, twins, intrauterine fetal death) and

(8) others (elective CS, unknown, post-date, prolonged premature rupture of membranes).

We modified Methods and Results according to the above mentioned revised indications.

Introduction

Line 93: anesthesia complications-related. I assume you mean anaesthesia-related complications?

Thank you for your suggestion, we revised accordingly.

Line 94: could you elaborate on the risk of incontinence and pelvic organ prolapse as complications of caesarean section?

Dear reviewer, thank you for seeking clarification on this matter, as we have identified an error in our description.

The source of our statement is: Sandall J, Tribe RM, Avery L, Mola G, Visser GH, Homer CS, Gibbons D, Kelly NM, Kennedy HP, Kidanto H, Taylor P, Temmerman M. Short-term and long-term effects of caesarean section on the health of women and children. Lancet. 2018 Oct 13;392(10155):1349-1357. doi: 10.1016/S0140-6736(18)31930-5. Quoting Sandall: “Long-term sequalae of CS include pelvic adhesions, small bowel obstruction, menorrhagia, dysmenorrhoea, chronic pain, sexual dysfunction, subfertility, urinary and faecal incontinence, and pelvic organ prolapse”.

However, my co-authors and I have realized that the connection between CS and urinary incontinence or pelvic organ prolapse is highly improbable. In the same cited article, the exact opposite is described a few lines later: “CS is associated with reduced risk of urinary incontinence, but the difference seems to level out with age” suggesting that the phrase contained in the original cited article may not be entirely accurate.

We have now removed 'risk of incontinence' and 'pelvic organ prolapse' from the list of possible complications, leaving only those confirmed by the literature.

Line 97: I would rephrase poor management, maybe into suboptimal management?

Thank you for your suggestion, we revised accordingly.

Methods

Line 170: does pre-eclampsia fall under extreme emergencies? Usually there is time to properly plan CS or induce labour? Same with placenta praevia (without blood loss), this is more often an elective caesarean section indication

Thank you for highlighing this particular aspects – all the conditions that were included in the classification were actually emergency conditions. The errror form our side was to not describe them in a proper manner.

As mentioned above, we have now revised the indications as “severe hypertensive disorder including severe preeclampsia” and “antepartum haemorragie due to abruptio placentae and placenta previa”

Line 172: would cord prolapse not fall under the extreme emergency caesarean sections?

We agree that cord prolapse was not categorized correctly. We have now removed cord prolapse from 'dystocia' and placed it within the 'intrapartum acute fetal distress' group. The recorded cases of cord prolapse were 3. We have modified both the methods and the results in accordance with this decision (Lines 2486-248) as well as Figure 1.

Results

Table 1: was the CS operator also the indicator? This would maybe even be of more importance, since many training programs focus on delivering skills (performing the procedure) but sometimes lack the teaching of proper diagnosing and decision-making

Thank you for this insightful comment; we greatly agree that this aspect is highly relevant, especially in the context of the PCMH, which has now become a teaching hospital. Unfortunately, we were unable to record this information, partly because it was not available in the medical records and also due to the impracticality of collecting it promptly for every cesarean section due to the high volume of deliveries.

We attempted to assess whether there were any differences in the percentages of cesarean sections performed by the two professional figures based on indications for cesarean section, but we did not observe any differences significant enough to warrant description (see figure below); therefore, we did not delve further into this topic.

We have included a comment in the discussion section to provide clarity on what was recorded:

In our study, over 70% of cesarean sections were performed by surgical technicians. However, unfortunately, it was not possible to record who made the decision to perform the cesarean section, and consequently, we are unable to assess the aspect of decision-making based on the different indications for cesarean section. This aspect likely warrants further investigation, especially considering that the PCMH has transitioned into a University Teaching Hospital and can now rely on mentors who can guide less skilled personnel.

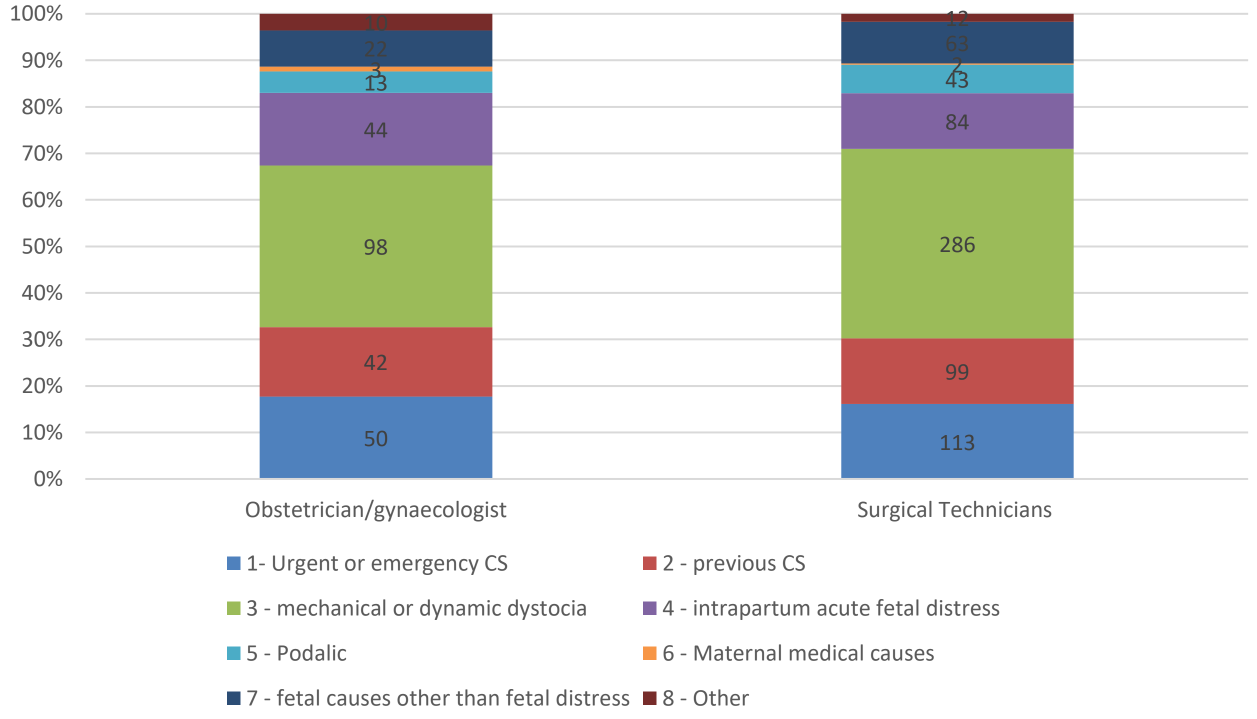

Line 217/218: I would for readibility explain briefly which women these groups entail.

Thank you for pointing out this potential improvement. We have now revised the sentence as follows:

Most women were in group 3, comprising multiparous women with a single term pregnancy in spontaneous labour (38.6% of the study population) and group 1, consisting of nulliparous women with a single term pregnancy in spontaneous labour (28.3%).

Discussion

Line 272: what guidelines suggest this ratio?

Thank you for pointing out this inaccuracy. We have now clarified the source and terms of reference of the ratio.

Additionally, we have revised the order of the discussion paragraphs, as that ratio was linked to the aspect of induction described just below. We have now merged the two paragraphs as follows:

The WHO Robson Implementation Manual suggest that the ratio between spontaneous and induced women (Group 1: Group 2) should be 2:1 or higher. In PCMH, the ratio is 7:1 which is extremely high and may suggests that an increase in the rate of inductions is expected to be beneficial. The overall rate of births with induction in our population was 3.1%

Line 288: how was dystocia defined?

The hospital staff uses the definitions from the National Guidelines of Sierra Leone. I'm copying here below as an example the page related ti the Unsatisfactory Progress of Labour

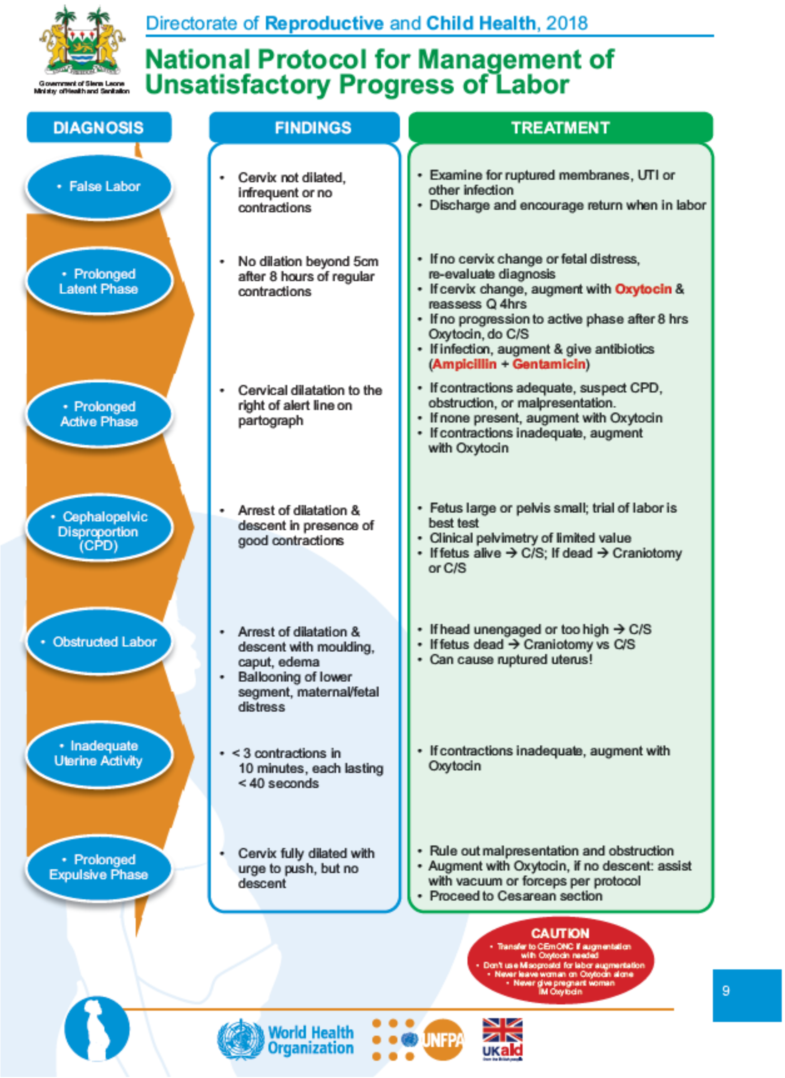

Although the data collection was partly conducted prospectively, most of the data on indications were obtained from medical records, which means the data collectors were unable to verify the application of guidelines in assigning indications for cesarean sections.

We have now added this specification in the Methods:

For each woman who underwent a CS, a single indication was reported from the hospital registry. Diagnosis and definition of all pathological conditions were derived from the National Protocol and Guidelines for Emergency and Newborn Care (21). The accuracy of the indications assigned in relation to the definitions was not verified by the data collectors.

Line 329: I would not consider this scope of this study so would omit this part (calcium supplementation)

Thank you for this suggestion, we agree with your comment and we now deleted that consideration from our discussion

What I generally miss in the discussion is elaboration on the shockingly high caesarean section rates for especially multigravida without caesarean section, and on the high volume of dystocia indications. Hereby is proper diagnosis of prolonged labour / dystocia crucial. I advise to provide more background as to how this was done in the hospital. How was labour progress monitored, who made indications, what could be reasons that caesarean section rates are this high. This will provide

opportunities for further research (which you highlighted) and stresses the urgency of tackling these caesarean section rates.

Thank you very much for this suggestion. In the discussion and conclusion sessions we now tried to explain more the context and some refllection on the CS rate.

As tertiary level and referral hospital for a large catchment area, PCMH receives many high-risk cases and obstetric complications. Despite the high CS rate registered at the hospital level, the CS rate at population level remains very low at less than 5% (33). In many low-resourced setting, the referral status, and therefore the emergencies received, contribute substantially to the CS rate (34). In PCMH, referred women accounted for more than 30% of total admissions in 2020 (20), and are likely to contribute substantially to the high CS rate within the hospital.

Improving the access and quality of antenatal care (39,40) as well as the appropriate utilization of partograph and intrapartum fetal monitoring are known as crucial strategies to prevent CS in women with dystocia (41). However, in PCMH, referrals of complicated deliveries from birth centers without capacity to perform CS contribute significantly to the dystocia diagnosis (34). The dramatic discrepancy between the CS rate in PCMH versus the population-based CS rate is consistent with this observation (15). We endorse the previous evidence calling for a nationwide effort aimed at increasing the availability of this life-saving procedure (15).

Reviewer: 1
Competing interests of Reviewer: No competing interest

Reviewer: 2
Competing interests of Reviewer: free from competing interests

Reviewer: 3
Competing interests of Reviewer: No conflict of interest.

Reviewer: 4
Competing interests of Reviewer: None

Reviewer: 5
Competing interests of Reviewer: None

VERSION 2 – REVIEW

**REVIEWER NAME**	Jauniaux, Eric
**REVIEWER AFFILIATION**	UCL, EGA Institute for Women Health
**REVIEWER CONFLICT OF INTEREST**	None to declare
**DATE REVIEW RETURNED**	10-Jun-2024

**GENERAL COMMENTS**	Dear authors, I am happy with the answers you have given to my and the corresponding changes you have made in the manuscript.

**REVIEWER NAME**	Jayawardane, Asanka

**REVIEWER AFFILIATION**	University of Colombo Faculty of Medicine, Obstetrics and Gynecology
**REVIEWER CONFLICT OF INTEREST**	I have no competing interests
**DATE REVIEW RETURNED**	06-Jun-2024

**GENERAL COMMENTS**	I wish to congratulate the authors for presenting this revised audit on a very important aspect in quality improvement in maternity care- especially in a resource limited setting. They have addressed the relevant concerns in the discussion. The study has revealed multiple aspects for quality improvement in delivery of care in their setting.<br><br>As stated by the authors (and others in similar settings- see ref) one key issue in conducting patient record based quality improvement studies and audits is lack of reliability in recorded data. Some authors have reported this even with prospective studies.<br>Ref.<br><br>1. Senanayake, Hemantha, et al. "Implementation of the WHO manual for Robson classification: an example from Sri Lanka using a local database for developing quality improvement recommendations." BMJ open 9.2 (2019): e027317.<br>2. Jayasundara, Chandana S., et al. "Validation of RobsApp-Audit tool for Caesarean Section Trends." (2023).<br><br>I would encourage authors to elaborate on missing data, data reliability and possible quality improvement measures applicable in their setting.

VERSION 2 – AUTHOR RESPONSE

Reviewer: 4

Dr. Asanka Jayawardane, University of Colombo Faculty of Medicine

Comments to the Author:

I wish to congratulate the authors for presenting this revised audit on a very important aspect in quality improvement in maternity care- especially in a resource limited setting. They have addressed the relevant concerns in the discussion. The study has revealed multiple aspects for quality improvement in delivery of care in their setting.

As stated by the authors (and others in similar settings- see ref) one key issue in conducting patient record based quality improvement studies and audits is lack of reliability in recorded data. Some authors have reported this even with prospective studies.

Ref.

1. Senanayake, Hemantha, et al. "Implementation of the WHO manual for Robson classification: an example from Sri Lanka using a local database for developing quality improvement recommendations." BMJ open 9.2 (2019): e027317.

2. Jayasundara, Chandana S., et al. "Validation of RobsApp-Audit tool for Caesarean Section Trends." (2023).

I would encourage authors to elaborate on missing data, data reliability and possible quality improvement measures applicable in their setting.

Thank you very much for highlighting this important aspect, which is crucial not only for the applicability of the Robson Classification but also for monitoring any improvement interventions that we may want to implement.

We had already emphasized within the text the need to improve and systematize the collection of important information for a more accurate interpretation of data, such as Referral Status and a standardized classification of indications.

We have now also added comments in the discussion regarding data availability (which we found to be very high in our context for the variables required for the Robson classification), emphasizing the necessity of making the periodic processing of this information routine and sustainable.

We have expressed these concepts at the beginning of the Discussion and in the Conclusions section with the following statements:

“The Robson Classification has proven to be easily applicable even in settings with high organizational complexity and a large number of births, such as PCMH. In fact, only 1.2% of the records of women who gave birth during the study period lacked the necessary information to allow its application. However, the need for volunteer intervention for dedicated data collection indicates that, for now, such evaluations are only being captured for research purposes, and the conditions are not yet in place for them to be performed routinely and continuously as recommended (29,30).”

“The evaluation of CS according to the Robson classification should be routinely and prospectively introduced into clinical practice to improve the quality of the information collected and enable the monitoring of quality improvement interventions.”

Reviewer: 3

Prof. Eric Jauniaux, UCL

Comments to the Author:

Dear authors, I am happy with the answers you have given to my and the corresponding changes you have made in the manuscript.

Thank you once again for the valuable contribution you have made to improve this manuscript.

Reviewer: 4

Competing interests of Reviewer: I have no competing interests

Reviewer: 3

Competing interests of Reviewer: None to declare
